# Public Perceptions and Determinants of Preference for Robotic-Assisted Surgery in the United Arab Emirates: Cross-Sectional Study

**DOI:** 10.2196/97975

**Published:** 2026-09-11

**Authors:** Yasir Ahmed Mohammed Elhadi, Aminu S Abdullahi, Abdelrahman Mohamed Alblooshi, Abdulla Alhosani, Arwa Al Khalidi, Mohammed Saleem, Shamma Rashed Mohammed Alrashdi, Reem Saleh Aljaberi, Azhar T Rahma

**Affiliations:** 1 Institute of Public Health, College of Medicine and Health Sciences, United Arab Emirates University, Tawam St, Al Ain, 15551, United Arab Emirates, 971 501126098; 2 College of Medicine and Health Sciences, United Arab Emirates University, Al Ain, United Arab Emirates; 3 Department of Statistics and Data Analytics, College of Business and Economics, United Arab Emirates University, Al Ain, United Arab Emirates; 4 Department of Biological Sciences, College of Medicine and Health Sciences, Khalifa University, Abu Dhabi, United Arab Emirates

**Keywords:** robot-assisted surgery, United Arab Emirates, health services research, human-robot interaction, technology adoption, technology acceptance, surgery

## Abstract

**Background:**

Robotic-assisted surgery (RAS) is increasingly being integrated into surgical services worldwide. Successful implementation of robotic surgical technologies depends not only on clinical effectiveness but also on public trust, perceptions of safety, and willingness to use technology-enabled care. In the United Arab Emirates, the adoption of RAS is expanding rapidly; however, evidence regarding public perceptions and factors influencing acceptance remains limited.

**Objective:**

This study aims to assess awareness, understanding, perceptions, and factors independently associated with preference for RAS among residents in the United Arab Emirates.

**Methods:**

A community-based cross-sectional survey was conducted between July and November 2025 among adults aged 18 years or older residing across multiple emirates in the United Arab Emirates. Participants were recruited using a nonprobability convenience sampling approach through hospitals, shopping malls, community events, university mailing lists, and social media platforms. A bilingual Arabic-English questionnaire adapted from previously validated robotic surgery perception instruments was administered. Descriptive statistics summarized participant responses. Associations between participant characteristics and perceptions of RAS were examined using chi-square and Fisher exact tests. To adjust for potential confounding, a multivariable Firth-penalized logistic regression model was fitted to identify factors independently associated with preference for RAS over traditional surgeon-performed surgery. Statistical analyses were conducted using R (version 4.4.1).

**Results:**

A total of 508 participants were included. Although 85% (n=432) had previously heard of RAS, only 38% (n=195) demonstrated an accurate functional understanding of robotic surgical systems. Overall, 33% (n=169) preferred RAS, while 35% (n=178) perceived robotic-assisted procedures as safe. The most frequently reported concerns were robotic malfunction (n=360, 71%), reduced human involvement (n=239, 47%), and procedural costs (n=183, 36%). Perceived benefits included improved surgical precision (n=331, 65%) and reduced complications (n=183, 36%). In multivariable analysis, participants who perceived RAS as safe had substantially higher odds of preferring a robot-assisted surgeon than those who were uncertain about its safety (adjusted odds ratio [aOR]=3.00, 95% CI 1.56‐5.87; *P*<.001). Conversely, participants who perceived RAS as unsafe had substantially lower odds of preferring a robot-assisted surgeon than those who were uncertain about its safety (aOR=0.08, 95% CI 0.02‐0.26; *P*<.001). No other factors were independently associated with surgery preference after adjustment.

**Conclusions:**

Within this predominantly highly educated convenience sample of United Arab Emirates residents, awareness of RAS was high, but accurate understanding and acceptance remained limited. Perceived safety emerged as the factor shaping preference for RAS, highlighting the importance of trust and confidence in technology-enabled health care. These findings suggest that public education, transparent communication regarding surgeon oversight, and patient engagement strategies may be important components of the successful implementation of robotic surgical services in the United Arab Emirates.

## Introduction

Robotic-assisted surgery (RAS) has emerged as a major advancement in minimally invasive surgical care, transforming operative practice across multiple specialties through enhanced precision, dexterity, visualization, and ergonomic support for surgeons [1,2]. Over the past 2 decades, the global adoption of robotic platforms has expanded rapidly in general surgery, urology, gynecology, cardiothoracic surgery, and neurosurgery, supported by technological innovations and the increasing integration of AI-enhanced capabilities into surgical systems [3,4]. Contemporary robotic systems increasingly incorporate digital interfaces, image-guided navigation, machine learning–supported decision assistance, and real-time data integration, positioning RAS as an important component of the broader digital transformation of health care [5-7]. Despite growing clinical adoption and evidence supporting several perioperative benefits, including reduced blood loss, shorter hospital stay, and improved surgical precision in selected procedures, public acceptance remains essential for the successful integration of robotic technologies into routine health care delivery [8,9].

Acceptance of emerging health care technologies is influenced not only by clinical effectiveness but also by human factors, including trust, perceived safety, transparency, perceived autonomy, and comfort with human-machine interaction [10-13]. The technology acceptance model and related frameworks emphasize that perceived usefulness, perceived ease of use, and trust strongly influence willingness to adopt novel technologies [14-16]. In the context of robotic surgery, concerns regarding machine autonomy, loss of human control, technical malfunction, depersonalization of care, and ethical implications may shape patient attitudes independently of objective clinical outcomes [17-19]. Public perceptions of AI-integrated technologies are also increasingly influenced by broader societal anxieties surrounding automation and decision-making by intelligent systems, particularly in high-risk settings such as health care [13,20-22]. Understanding these human-factor determinants is therefore critical for ensuring patient-centered implementation and equitable access to advanced surgical technologies.

Existing international evidence demonstrates substantial variability in public awareness, understanding, and acceptance of RAS across different populations and health care systems. Studies from the United States and Europe have reported moderate public awareness but persistent misconceptions regarding robotic autonomy and surgeon involvement during robotic procedures [13,23]. Surveys conducted in Saudi Arabia, Kuwait, and Jordan similarly identified limited functional understanding of RAS, concerns regarding safety and technical failure, and demographic differences in acceptance, particularly across age and gender groups [10,24-27]. A recent scoping review further highlighted that public perceptions of robotic surgery are commonly characterized by uncertainty, limited trust, and confusion regarding the role of the surgeon during robotic procedures [28]. Although several studies reported positive perceptions regarding precision and innovation, many participants remained reluctant to personally undergo robotic-assisted procedures due to concerns about safety, cost, and reduced human involvement [23,24,28].

In the United Arab Emirates, rapid investment in digital health infrastructure and advanced medical technologies has accelerated the implementation of robotic surgical systems within tertiary health care centers [29]. Robotic-assisted procedures are increasingly performed across major public and private hospitals, reflecting the country’s broader health care innovation agenda and strategic emphasis on AI integration within health services. Nevertheless, evidence regarding public perceptions of RAS in the United Arab Emirates remains limited. Existing studies have primarily focused on health care professionals and medical students rather than the general public [30,31]. Earlier research examining public attitudes toward robotic surgery in the United Arab Emirates reported skepticism and limited awareness; however, this study was conducted more than a decade ago and may no longer reflect contemporary perceptions within the context of rapidly evolving health care technologies and increased national investment in digital medicine [32]. This study aimed to examine public awareness, understanding, perceptions, and preference for RAS among residents in the United Arab Emirates. Specifically, the study sought to (1) assess public awareness and functional understanding of RAS; (2) examine perceived benefits, risks, and trust-related concerns associated with RAS; and (3) identify demographic and experiential factors associated with preference for RAS and perceptions of safety.

## Methods

### Study Design and Setting

A community-based cross-sectional survey was conducted between July and November 2025 across multiple emirates in the United Arab Emirates, including Abu Dhabi, Dubai, Sharjah, Ajman, Ras Al-Khaimah, Fujairah, and Umm Al-Quwain. The study was reported in line with the STROBE (Strengthening the Reporting of Observational Studies in Epidemiology) recommendations [33].

### Participants and Sampling

Eligible participants were adults aged 18 years or older residing in the United Arab Emirates who were able to read either Arabic or English. Individuals professionally involved in robotics, AI, biomedical engineering, robotic systems development, or related technical fields were excluded to minimize expert-knowledge bias and ensure that responses reflected perceptions of the general public. A nonprobability convenience sampling approach was used. Participants were recruited through both in-person and online approaches to maximize demographic and geographic diversity. In-person recruitment was conducted in hospitals, shopping malls, community centers, and public events across multiple emirates. The online survey was disseminated through university mailing lists and social media platforms, including WhatsApp, Instagram, LinkedIn, and X (formerly Twitter).

The optimal required sample size was estimated using the single population proportion formula:


$$n=\frac{Z^{2}P(1-P)}{d^{2}}$$

Assuming a 50% expected prevalence, a 95% confidence level, a 5% margin of error, and a z score of 1.96. The calculated minimum sample size was 384 participants. To account for incomplete responses and potential nonresponse, the recruitment target was increased, and a final sample of 508 participants was included in the analysis.

### Data Collection Tool

The bilingual (Arabic-English) questionnaire was adapted from previously validated RAS perception surveys conducted in the Gulf region and internationally [24,27,32]. The instrument underwent expert review by specialists in public health, digital health, and RAS to assess content validity, clarity, and cultural appropriateness for the UAE context. Minor modifications in wording and terminology were implemented following expert feedback to improve comprehensibility and contextual relevance.

Translation of the questionnaire followed the World Health Organization forward-backward translation methodology [34]. The English version was initially translated into Arabic by bilingual researchers and was then independently back-translated into English to ensure semantic equivalence and conceptual consistency between versions. Pilot testing was conducted among 20 bilingual UAE residents representing the target population to evaluate readability, comprehension, cultural appropriateness, and survey flow. Feedback obtained during pilot testing was used to refine question wording and improve usability. Pilot participants were excluded from the final analysis. The questionnaire included domains assessing demographic characteristics, technology experience, awareness and understanding of RAS, perceptions and attitudes toward RAS, and prior experiences with robotic surgery. The perception and attitude items demonstrated acceptable internal consistency within the study sample (Cronbach *α*=0.81).

### Measures

The questionnaire consisted primarily of closed-ended items divided into five domains: (1) demographic characteristics, (2) technology experience, (3) awareness and understanding of RAS, (4) perceptions and attitudes toward RAS, and (5) patient-reported experiences among participants with prior exposure to robotic surgery. Demographic variables included age, gender, nationality, educational attainment, occupation, marital status, monthly income, health insurance status, chronic disease status, and medical travel history. Awareness and understanding of RAS were assessed using items evaluating prior exposure to robotic surgery, information sources, and functional understanding of robotic systems. Participants were classified as having “good understanding” if they correctly identified that robotic surgery involves a surgeon operating robotic arms from a control console. Perceptions and attitudes toward RAS included perceived safety, trust in robotic technologies, perceived benefits and risks, willingness to undergo RAS, concerns regarding robotic malfunction or loss of human control, and beliefs regarding the future role of robotic surgery in health care.

### Ethical Considerations

This study was conducted in accordance with the research standards outlined in the Declaration of Helsinki. Ethical approval was obtained from the Social Sciences Research Ethics Committee at United Arab Emirates University (reference number: ERSC_2025_5861). All participants received an information sheet explaining the study objectives, the voluntary nature of participation, anonymity, and confidentiality measures. Electronic or written informed consent was obtained before participation. Participation was voluntary, and respondents were assured of their right to withdraw at any time without consequence. No identifiable personal information was collected. All data were collected anonymously and handled in accordance with ethical research standards. Participants were not financially compensated for participation.

### Statistical Analysis

Missing data were minimal (<2% across all variables). Participants with missing data for variables included in a specific analysis were excluded only from that analysis. Data were analyzed using the R statistical software (version 4.4.1; R Foundation for Statistical Computing). Categorical variables were summarized using frequencies and percentages. Exact binomial 95% CIs were calculated for key proportions.

The variable assessing participants’ understanding of RAS included 5 response options. Participants were classified as having a good understanding of RAS only if they correctly identified that robotic surgery involves a surgeon sitting at a control unit and operating robotic arms. All other responses, including “I don’t know,” were classified as limited understanding. For the ease of interpretation in inferential analyses, age was recategorized from 6 original categories (18‐24, 25‐34, 35‐44, 45‐54, 55‐64, and ≥65 y) into 2 groups (18‐44 y and ≥45 y).

Associations between participants’ characteristics and 2 key outcomes, including preference for and perceived safety of RAS, were assessed using the chi-square test or Fisher exact test, as appropriate. Variables examined included demographic characteristics, health insurance status, chronic disease status, medical travel history, parental status, awareness of RAS, familiarity with individuals who had undergone robotic surgery, perceived availability of RAS in the UAE, sources of information about RAS, understanding of RAS, comfort with modern technology, and frequency of technology use. Statistical significance was defined as a 2-sided *P* value <.05. To adjust for potential confounding, a multivariable Firth-penalized logistic regression model was fitted to identify factors independently associated with preference for RAS. This method was used to address sparse data and potential quasi-complete separation [35]. The primary outcome was preference for RAS versus traditional surgeon, as participants with no preference were excluded from the primary analysis. Variables were selected a priori based on theoretical relevance and included gender, age, nationality, monthly income, knowing someone who had undergone RAS, understanding of RAS, and perceived safety of RAS. Adjusted odds ratios (aORs) and 95% CIs were reported. To assess the robustness of the findings, a sensitivity analysis was performed by redefining the outcome as preference for a robot-assisted surgeon versus all other responses (traditional surgeon or no preference) using the same multivariable Firth-penalized logistic regression model.

## Results

### Participant Recruitment and Characteristics

A total of 508 participants were included in the final analysis. The study sample comprised 57% (n=291) female participants and 55% (n=282) Emiratis (Table 1). Most participants (n=328, 65%) were aged 18‐44 years, while 35% (n=180) were aged 45 years or older. The majority (n=316, 62%) resided in the Abu Dhabi Emirate, held a bachelor’s degree or higher qualification (n=402, 79%), were married (n=317, 62%), and had children (n=322, 63%). Approximately one-third of participants worked in health care–related fields, including 20% who identified as health care professionals. Regarding monthly income, 31% (n=157) reported earning more than 20,000 AED (United Arab Emirates dirham; 1 AED=0.27 USD as of August 4, 2026 ) per month, while 13% (n=64) reported earning less than 5000 AED per month. Chronic medical conditions were reported by 22% (n=113) of the participants, 29% (n=145) reported having health insurance coverage, and 16% (n=79) reported a history of medical travel.

**Table 1.** Participants’ characteristics (N=508).

Characteristic	Participants, n (%)
Gender	
Female	291 (57.3)
Male	217 (42.7)
Age (y)	
18‐24	102 (20.1)
25‐34	103 (20.3)
35‐44	123 (24.2)
45‐54	94 (18.5)
55‐64	72 (14.2)
≥65	14 (2.8)
Nationality	
Emirati	282 (55.5)
Non-Emirati	226 (44.5)
Residence	
Abu Dhabi	316 (62.2)
Ajman	16 (3.2)
Al-Fujairah	11 (2.2)
Dubai	92 (18.1)
Ras Al-Khaimah	12 (2.4)
Sharjah	59 (11.6)
Umm Al-Quwain	2 (0.3)
Education	
High school	68 (13.4)
Diploma	38 (7.5)
Bachelor’s degree	270 (53.1)
Master’s degree	132 (26)
Work field	
Business or administration	149 (29.3)
Education	58 (11.4)
Engineering	73 (14.4)
Medical or health care	160 (31.5)
Others	68 (13.4)
Marital status	
Divorced	22 (4.3)
Married	317 (62.4)
Single	167 (32.9)
Widow	2 (0.4)
Have children	322 (63.4)
Health care worker	100 (19.7)
Monthly income (AED^a^)	
<5000	64 (12.6)
5000‐9000	51 (10)
10,000‐20,000	100 (19.7)
>20,000	157 (30.9)
Prefer not to answer	136 (26.8)
Have a chronic condition	113 (22.2)
Medical travel (yes)	79 (15.6)
Have insurance coverage	145 (28.5)

a AED: United Arab Emirates dirham.

### Perceived Safety of RAS

Overall, 35% of the participants (95% CI 31%-39%) perceived RAS as safe, whereas 22% (95% CI 19%‐26%) considered it unsafe, and 43% (95% CI 38%‐47%) reported uncertainty regarding its safety (Table S1 in Multimedia Appendix 1). Several participant characteristics were significantly associated with perceptions of safety. Male participants were more likely than female participants to perceive RAS as safe (50% vs 24%; *P*<.001). Participants aged 45 years or older were also more likely to perceive robotic surgery as safe compared with younger adults aged 18‐44 years (42% vs 31%; *P*=.01). Emirati nationality, higher monthly income, and health care worker status were also associated with more favorable safety perceptions. Participants with health insurance coverage were substantially more likely to perceive RAS as safe than uninsured participants (51% vs 29%; *P*<.001). Participants reporting chronic medical conditions also demonstrated more positive safety perceptions compared with those without chronic conditions (41% vs 33%; *P*=.04).

Prior exposure and understanding strongly influenced safety perceptions. Participants who knew someone who had undergone robotic surgery were more likely to perceive RAS as safe than those without such exposure (66% vs 36%; *P*<.001). Similarly, participants who had previously heard of robotic surgery (38% vs 16%; *P*<.001), demonstrated accurate functional understanding of RAS (42% vs 31%; *P*=.02), or preferred robotic-assisted procedures over traditional surgery (58% vs 16%; *P*<.001) were significantly more likely to report positive perceptions regarding safety. Participants exposed to information regarding robotic surgery through television, newspapers, medical websites, medical conferences or seminars, health care professionals, or personal experience demonstrated significantly greater confidence in the safety of RAS than those without exposure through these channels. Educational attainment, having children, medical travel history, comfort with modern technology, frequency of technology use, and learning about robotic surgery through social media or university lectures were not significantly associated with perceived safety.

### Perceptions Regarding the Future Role and Precision of RAS

Most participants expressed optimism regarding the future role of RAS within health care systems. Overall, 67% (n=340) believed that robotic surgery would eventually become a standard surgical approach, while 61% (n=310) perceived robotic systems to be more precise than human surgeons. Despite these favorable perceptions regarding technological advancement and precision, the willingness to personally undergo robotic-assisted procedures remained limited. Only 37% (n=188) reported that they would choose RAS if it represented the only available treatment option, whereas 31% (n=158) stated that they would still decline robotic-assisted procedures under such circumstances. Additional perception-related responses are presented in Table S2 in Multimedia Appendix 1.

### Perceived Benefits and Concerns Regarding RAS

Participants reported both perceived benefits and concerns regarding RAS. The most frequently reported perceived benefit was improved surgical precision (n=331, 65%), followed by reduced complications and surgical risks (n=183, 36%). Additionally, 20% (n=102) believed RAS could reduce postoperative pain, while 20% (n=102) associated robotic surgery with faster postoperative recovery. Concerns regarding RAS were widespread, with 94% (n=478) of the participants reporting at least one concern related to robotic surgical systems. The most commonly reported concern was the possibility of a robotic malfunction during surgery (n=360, 71%). Additional concerns included reduced involvement of human surgical skills (n=239, 47%), high procedural costs (n=183, 36%), and ethical or technical concerns related to robotic technologies (n=133, 26%).

### Perceived Suitability of RAS Across Surgical Specialties

Participants identified several surgical specialties they perceived as particularly suitable for RAS. General surgery was the most frequently identified specialty (n=218, 43%), followed by cardiac surgery (n=188, 37%) and neurosurgery (n=168, 33%). Additional specialties perceived as appropriate for robotic-assisted procedures included orthopedic surgery (n=152, 30%), urological surgery (n=147, 29%), and cardiothoracic surgery (n=97, 19%).

### Preferences for RAS and Associated Factors

Among the 508 participants, 33% (95% CI 29%‐38%) preferred RAS, 50% (95% CI 45%‐54%) preferred traditional surgeon-performed procedures, and 17% (95% CI 14%‐21%) reported no preference (Table 2). The distribution of surgery preference differed significantly by gender (*P*<.001), age (*P*<.001), nationality (*P*<.001), monthly income (*P*=.002), having children (*P*=.023), health insurance coverage (*P*<.001), and perceived safety of RAS (P<.001). The proportion preferring RAS was higher among male than female participants (40% vs 29%), participants aged 45 years or older than those aged 18–44 years (46% vs 27%), Emirati than non-Emirati participants (39% vs 26%), participants with a monthly income of 20,000 AED or more than those with lower incomes (41% vs 28%), participants with children than those without children (37% vs 26%), and among participants with health insurance coverage than among those without coverage (42% vs 23%).

Prior awareness and exposure to robotic surgery were also significantly related to the distribution of surgery preference. The distribution differed significantly according to whether participants knew someone who had undergone RAS (*P*<.001), had previously heard of robotic surgery (*P*<.001), their functional understanding of RAS (P=.002), and whether they believed that RAS was available in the United Arab Emirates (*P*<.001). The proportion preferring RAS was higher among participants who knew someone who had undergone RAS than among those who did not (60% vs 32%), those who had previously heard of robotic surgery than among those who had not (36% vs 18%), those with good rather than poor functional understanding of RAS (35% vs 32%), and those who believed that RAS was available rather than unavailable in the United Arab Emirates (46% vs 27%). The distribution of surgery preference also differed significantly according to whether participants had learned about RAS through television (*P*=.027), social media (*P*=.009), or personal experience (*P*=.012). The corresponding proportions preferring RAS among participants with versus without exposure through these channels were 37% versus 32%, 37% versus 29%, and 57% versus 32%, respectively.

**Table 2.** Preferences for surgery and participants’ characteristics^a^.

Characteristic	Surgery preference			
	Robot-assisted surgeon (n=169)	Traditional surgeon (n=253)	No preference (n=86)	*P* value
Overall (95% CI)	33% (29%‐38%)	50% (45%‐54%)	17% (14%‐21%)	—^b^
Gender, n (%)				<.001
Female	83 (29)	171 (59)	37 (13)	
Male	86 (40)	82 (38)	49 (23)	
Age (y), n (%)				<.001
18‐44	87 (27)	183 (56)	58 (18)	
≥45	82 (46)	70 (39)	28 (16)	
Nationality, n (%)				<.001
Emirati	110 (39)	115 (41)	57 (20)	
Non-Emirati	59 (26)	138 (61)	29 (13)	
Children, n (%)				.02
Yes	120 (37)	155 (48)	47 (15)	
No	49 (26)	98 (53)	39 (21)	
Monthly income (AED^c^), n (%)				.002
<20,000	61 (28)	125 (58)	29 (13)	
≥20,000	64 (41)	62 (39)	31 (20)	
Have insurance coverage, n (%)				<.001
Yes	61 (42)	49 (34)	35 (24)	
No	22 (23)	58 (62)	14 (15)	
Not sure	86 (32)	146 (54)	37 (14)	
Know someone who had RAS^d^, n (%)				<.001
Yes	28 (60)	13 (28)	6 (13)	
No	114 (32)	180 (50)	67 (19)	
Heard of RAS, n (%)				<.001
Yes	155 (36)	199 (46)	78 (18)	
No	14 (18)	54 (71)	8 (11)	
Learned about RAS from television, n (%)				.03
Yes	53 (37)	59 (41)	32 (22)	
No	116 (32)	194 (53)	54 (15)	
Learned about RAS from social media, n (%)				.009
Yes	107 (37)	129 (44)	57 (19)	
No	62 (29)	124 (58)	29 (13)	
Learned about RAS from personal experience, n (%)				.01
Yes	13 (57)	5 (22)	5 (22)	
No	156 (32)	248 (51)	81 (17)	
Understanding of RAS, n (%)				.002
Good	68 (35)	81 (42)	46 (24)	
Limited	101 (32)	172 (55)	40 (13)	
Believes RAS is available in the United Arab Emirates, n (%)				<.001
Yes	91 (46)	69 (35)	38 (19)	
No	4 (27)	10 (67)	1 (6.7)	
Not sure	74 (25)	174 (59)	47 (16)	
Believes RAS is safe, n (%)				<.001
Yes	98 (56)	40 (22)	40 (22)	
No	6 (5.5)	103 (91)	4 (3.5)	
Not sure	65 (30)	110 (51)	42 (19)	

a Other factors explored but not found to be significantly correlated with surgery preferences include education, health care worker, having a chronic condition, medical travel, comfort level with using modern technology, frequency of technology use, other ways of learning about RAS— newspapers, medical websites, medical conferences or seminars, health care professionals, university or academic lectures, and other sources.

b Not applicable.

c AED: United Arab Emirates dirham.

d RAS: robotic-assisted surgery.

**Figure 1.**
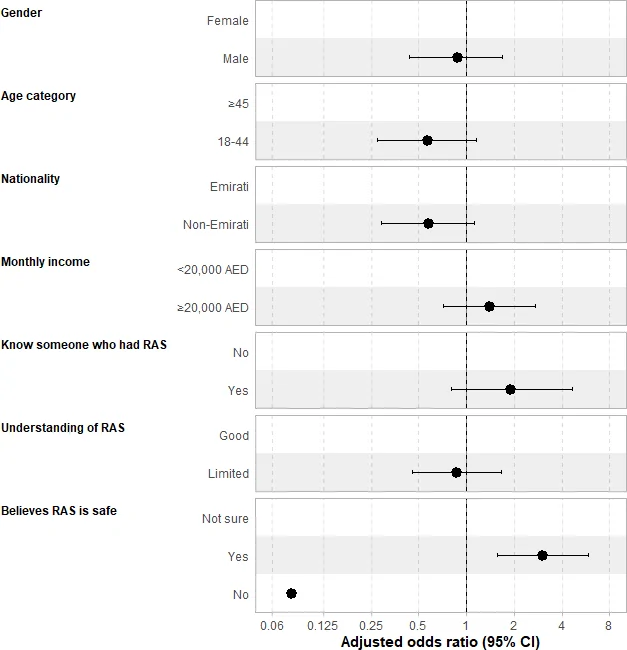
Multivariable logistic regression analysis of factors associated with preference for a robot-assisted surgeon over a traditional surgeon. Odds ratios are adjusted for all variables shown. Error bars represent 95% CIs. The dashed vertical line denotes the null value (odds ratio=1). RAS: robotic-assisted surgery.

In the adjusted analysis using Firth penalized logistic regression, perceived safety of RAS was the only factor independently associated with preference for a robot-assisted surgeon over a traditional surgeon (Figure 1). Participants who perceived RAS as safe had substantially higher odds of preferring a robot-assisted surgeon than those who were uncertain about its safety (aOR=3.00, 95% CI 1.56‐5.87; *P*<.001). Conversely, participants who perceived RAS as unsafe had substantially lower odds of preferring a robot-assisted surgeon than those who were uncertain about its safety (aOR=0.08, 95% CI 0.02‐0.26; *P*<.001). No other factors were independently associated with surgery preference after adjustment.

A sensitivity analysis redefining the outcome as preference for a robot-assisted surgeon versus all other responses (traditional surgeon or no preference) yielded materially similar findings (Table S3 in Multimedia Appendix 1). Perceived safety remained the only consistent independent factor associated with preference, although younger age (18‐44 y) also reached statistical significance in the sensitivity analysis.

## Discussion

### Principal Findings

This study provides insights into perceptions of RAS among a convenience sample of predominantly highly educated residents in the United Arab Emirates. Although most participants had previously heard of RAS, only a minority demonstrated an accurate functional understanding of surgeon control and robotic operation. In this sample, acceptance of robotic-assisted procedures was relatively limited: only one-third of the participants preferred RAS to conventional surgery, and approximately one-third perceived robotic surgery as safe. Concerns regarding robotic malfunction, reduced human involvement, and technical reliability were common. Nevertheless, many participants simultaneously recognized potential benefits of robotic surgery, particularly improved surgical precision and reduced complications, highlighting an important tension between perceived innovation and perceived risk. Importantly, perceived safety emerged as the strongest independent factor associated with preference for RAS. Participants who believed RAS was safe were substantially more likely to prefer robotic-assisted procedures, even after adjustment for demographic and experiential factors. These findings suggest that trust and confidence in the safety of robotic surgical technologies may play a more important role in acceptance than most demographic characteristics.

The findings of this study are broadly consistent with international literature demonstrating limited public understanding and cautious acceptance of RAS despite increasing global adoption of robotic technologies in health care [28,36,37]. RAS across multiple countries is frequently characterized by uncertainty, misconceptions regarding robotic autonomy, and limited trust in machine-assisted procedures [37]. Similar findings have been reported in studies from the United States, Europe, and Gulf countries, where awareness of robotic surgery may be moderate, but functional understanding remains poor [28,38-41].

In the present study, many participants expressed uncertainty about the surgeon’s role during robotic procedures, with misconceptions about robotic autonomy and machine decision-making remaining common. These findings align with previous surveys reporting that large proportions of the public incorrectly believe that robotic systems operate independently of surgeons or possess autonomous surgical capabilities [23,25,26,42]. Such misconceptions likely reflect broader societal anxieties regarding AI-driven technologies and automation in health care settings [12,13]. These findings are consistent with the concept that acceptance of robotic surgery may be influenced not only by perceived clinical outcomes but also by trust, transparency, and understanding of human oversight within technologically mediated care [15,16].

Despite recognizing potential benefits of robotic surgery, participants in this study demonstrated relatively cautious willingness to personally undergo robotic-assisted procedures. Similar patterns have been observed internationally [17,24,36,40]. Previous studies have shown that patients may perceive robotic surgery as technologically advanced yet remain hesitant to accept it for their own treatment [17,36]. This apparent contradiction likely reflects underlying concerns regarding safety, reliability, and perceived loss of human control during surgery. In the present study, concerns regarding robotic malfunction represented the most frequently reported barrier to acceptance, consistent with previous studies identifying technical failure and loss of surgeon control as major public concerns surrounding RAS [17,18,23].

Participants in this study also perceived robotic surgery as associated with improved surgical precision and better clinical outcomes, including reduced complications, lower postoperative pain, and faster recovery. These findings align with previous international and regional studies reporting that the public commonly associates robotic-assisted procedures with enhanced precision and minimally invasive surgical benefits [25,28,42,43]. However, these perceived advantages were often offset by concerns about safety, ethical implications, procedural costs, and reduced human interaction in care. Similar concerns regarding depersonalization of care and diminished surgeon presence have been reported in qualitative studies examining patient perceptions of robotic-assisted procedures [38,44].

The present findings suggest that human factors play a central role in shaping public acceptance of RAS. Trust, perceived safety, understanding of surgeon oversight, and familiarity with robotic technologies were strongly associated with acceptance of RAS in bivariate analyses. Participants with prior exposure to robotic surgery, either through personal experience or knowing someone who had undergone robotic-assisted procedures, were substantially more likely to perceive RAS as safe and preferable to conventional surgery. This finding is consistent with previous evidence showing that familiarity and direct exposure are associated with lower uncertainty and greater trust in emerging medical technologies [28,36].

The study findings indicate that media exposure may help shape perceptions of RAS. Many participants reported learning about robotic surgery primarily through television, social media, internet sources, or word of mouth rather than directly from health care professionals. Similar patterns have been reported internationally, particularly in Middle Eastern populations [42,43]. Although media exposure may improve awareness of robotic technologies, inaccurate or sensationalized portrayals of robotic surgery may also contribute to fear, misunderstanding, and exaggerated perceptions of risk [45].

Public misconceptions regarding robotic autonomy and technical failure may therefore partly reflect the quality and framing of publicly available information regarding robotic surgery. Previous studies have demonstrated that targeted educational interventions and physician-led explanations can substantially improve patient understanding and confidence regarding robotic-assisted procedures [36,39].

Importantly, the multivariable analysis demonstrated that perceived safety was the only independent factor associated with preference for RAS. Participants who believed RAS was safe were substantially more likely to prefer robotic-assisted procedures than traditional surgeon-performed surgery, even after adjustment for demographic and experiential factors. In contrast, most demographic characteristics lost statistical significance after adjustment. These findings suggest that trust in the safety of robotic systems may be more influential than demographic characteristics in determining acceptance of robotic-assisted medical procedures.

From a health services research perspective, these findings have important implications for the implementation and adoption of technology-enabled surgical services. Successful integration of RAS into routine clinical practice may depend not only on technological capability and clinical effectiveness but also on addressing patient concerns regarding safety, reliability, and human oversight. Implementation strategies that strengthen public trust and improve understanding of surgeon control during robotic procedures may therefore play a critical role in promoting acceptance and uptake of robotic-assisted surgical services.

### Limitations

This study has several limitations. First, the use of nonprobability convenience sampling may have introduced selection bias and limited the generalizability of the findings. Second, the sample included a disproportionately high proportion of participants with bachelor’s degrees or higher qualifications and a substantial proportion working in health care–related fields, which may not reflect the demographic composition of the broader United Arab Emirates’ population. Third, the cross-sectional design precludes causal inference regarding factors associated with perceptions of RAS. Finally, all data were self-reported and may be subject to recall and social desirability biases. Therefore, the findings should be interpreted as reflecting perceptions within this study sample rather than the UAE population.

## Supplementary material

10.2196/97975 Multimedia Appendix 1 Supplementary tables.

## Abbreviations

AED: United Arab Emirates dirham
aOR: adjusted odds ratio
RAS: robotic-assisted surgery
STROBE: Strengthening the Reporting of Observational Studies in Epidemiology
