# Supplementary material for: Public Perceptions and Determinants of Preference for Robotic-Assisted Surgery in the United Arab Emirates: Cross-Sectional Study
Source: JMIR Hum Factors. 2026 Sep 11;13:e97975. doi: 10.2196/97975 (PMC13566074; doi:10.2196/97975)
Supplement: Supplementary file 1 [file humanfactors-v13-e97975-s001.docx]

**Supplementary tables**

**Table S1: Belief in the safety of robot-assisted surgery by participants’ characteristics**

| **Characteristic** | **Believe that robot-assisted surgery is safe** | | | **p-value** |
| --- | --- | --- | --- | --- |
|  | **Yes** (N=178) | **No** (N=113) | **Not sure** (N=217) |  |
| **Overall (95% CI)** | 35% (31–39) | 22% (19–26) | 43% (38–47) | — |
| **Gender** |  |  |  | <.001 |
| Female | 70 (24) | 88 (30) | 133 (46) |  |
| Male | 108 (50) | 25 (12) | 84 (39) |  |
| **Age** |  |  |  | .003 |
| 18–44 | 102 (31) | 87 (27) | 139 (42) |  |
| ≥45 | 76 (42) | 26 (14) | 78 (43) |  |
| **Nationality** |  |  |  | .023 |
| Emirati | 106 (38) | 50 (18) | 126 (45) |  |
| Non-Emirati | 72 (32) | 63 (28) | 91 (40) |  |
| **Healthcare worker** |  |  |  | .034 |
| Yes | 46 (46) | 17 (17) | 37 (37) |  |
| No | 132 (32) | 96 (24) | 180 (44) |  |
| **Monthly income** |  |  |  | .002 |
| <20,000 AED | 63 (29) | 52 (24) | 100 (47) |  |
| ≥20,000 AED | 73 (46) | 25 (16) | 59 (38) |  |
| **Have insurance coverage** |  |  |  | <.001 |
| Yes | 74 (51) | 17 (12) | 54 (37) |  |
| No | 27 (29) | 30 (32) | 37 (39) |  |
| Not sure | 77 (29) | 66 (25) | 126 (47) |  |
| **Have chronic condition** |  |  |  | 0.003 |
| Yes | 46 (41) | 12 (11) | 55 (49) |  |
| No | 132 (33) | 101 (26) | 162 (41) |  |
| **Know someone who had RAS** |  |  |  | <.001 |
| Yes | 31 (66) | 2 (4.3) | 14 (30) |  |
| No | 130 (36) | 82 (23) | 149 (41) |  |
| **Heard of RAS** |  |  |  | <.001 |
| Yes | 166 (38) | 81 (19) | 185 (43) |  |
| No | 12 (16) | 32 (42) | 32 (42) |  |
| **Learned about RAS through television** |  |  |  | .047 |
| Yes | 58 (40) | 22 (15) | 64 (44) |  |
| No | 120 (33) | 91 (25) | 153 (42) |  |
| **Learned about RAS through newspaper** |  |  |  | .002 |
| Yes | 29 (54) | 4 (7.4) | 21 (39) |  |
| No | 149 (33) | 109 (24) | 196 (43) |  |
| **Learned about RAS from personal experience** |  |  |  | .015 |
| Yes | 14 (61) | 1 (4.3) | 8 (35) |  |
| No | 164 (34) | 112 (23) | 209 (43) |  |
| **Learned about RAS from medical websites** |  |  |  | .005 |
| Yes | 57 (47) | 24 (20) | 40 (33) |  |
| No | 121 (31) | 89 (23) | 177 (46) |  |
| **Learned about RAS from medical conferences** |  |  |  | .001 |
| Yes | 42 (51) | 18 (22) | 22 (27) |  |
| No | 136 (32) | 95 (22) | 195 (46) |  |
| **Learned about RAS from healthcare professional** |  |  |  | .026 |
| Yes | 53 (45) | 20 (17) | 44 (38) |  |
| No | 125 (32) | 93 (24) | 173 (44) |  |
| **Understanding of RAS** |  |  |  | .030 |
| Good | 82 (42) | 37 (19) | 76 (39) |  |
| Limited | 96 (31) | 76 (24) | 141 (45) |  |
| **Preference** |  |  |  | <.001 |
| RAS | 98 (58) | 6 (3.6) | 65 (38) |  |
| Traditional surgeon | 40 (16) | 103 (41) | 110 (43) |  |
| No preference | 40 (47) | 4 (4.7) | 42 (49) |  |
| **Believes RAS is available in the UAE** |  |  |  | <0.001 |
| Yes | 105 (53) | 17 (8.6) | 76 (38) |  |
| No | 4 (27) | 9 (60) | 2 (13) |  |
| Not sure | 69 (23) | 87 (29) | 139 (47) |  |

Values are presented as n(%) unless indicated elsewhere. Other factors explored but not significantly correlated with belief in RAS safety include education, having children, medical travel, comfort level with using modern technology, frequency of technology use, and other ways of learning about RAS—social media, university or academic lectures, and other sources.

**Table S2: Summary of responses to additional perception- and attitude-related questions**

| **Questions** | **N** | **%** |
| --- | --- | --- |
| **Have you heard of robotic-assisted surgery before? (yes)** | 432 | 85 |
| **If yes, from what source(s) did you learn about it?** |  |  |
| Television | 144 | 28 |
| Social media | 293 | 58 |
| Newspapers/magazines | 54 | 11 |
| Medical websites | 121 | 24 |
| Medical conferences or seminars | 82 | 16 |
| Healthcare professionals | 117 | 23 |
| University or academic lectures | 75 | 15 |
| Personal experience | 23 | 4.5 |
| Others | 13 | 2.6 |
| **What do you understand about robotic-assisted surgery?** |  |  |
| The robot follows the surgeon’s step-by-step instructions | 56 | 11 |
| The robot performs the surgery while the surgeon sits beside to ensure the safety of the patient | 112 | 22 |
| The surgeon programs the robot, and the robot performs the surgery | 66 | 13 |
| The surgeon sits at a control unit and moves the robot’s arms | 195 | 38 |
| I don't know | 79 | 16 |
| **What comes to mind when you hear the term "robotic-assisted surgery"?** |  |  |
| Less invasive than open surgery | 110 | 22 |
| Fewer complications than open surgery | 117 | 23 |
| Faster recovery than open surgery | 231 | 45 |
| More expensive than open surgery | 26 | 5.1 |
| More complications than open surgery | 26 | 5.1 |
| Slower than open surgery | 38 | 7.5 |
| Risk of robot malfunction during surgery | 221 | 44 |
| Risk of surgical errors due to robotics | 163 | 32 |
| Others | 19 | 3.7 |
| **Do you think robotic surgeons have more or less skill than traditional surgeons?** |  |  |
| Less skilled than traditional surgeons | 57 | 11 |
| More skilled than traditional surgeons | 198 | 39 |
| Same skill level as traditional surgeons | 121 | 24 |
| Not sure | 132 | 26 |
| **Compared to traditional surgery, do you think hospitals offering robotic-assisted surgery provide better or worse services?** |  |  |
| Better | 232 | 46 |
| Similar | 129 | 25 |
| Worse | 18 | 3.5 |
| Not sure | 129 | 25 |
| **Should all hospitals invest in robotic-assisted surgery?** |  |  |
| No, because it is expensive and not necessary | 107 | 21 |
| Yes, because it is an advanced and beneficial technology | 267 | 53 |
| Not sure | 134 | 26 |
| **How would you describe your personal experience with robotic-assisted surgery?** |  |  |
| Excellent | 30 | 6.3 |
| Very Good | 18 | 3.8 |
| Good | 17 | 3.6 |
| Bad | 1 | 0.2 |
| Not applicable | 407 | 86 |
| **Why did you choose this type of surgery?** |  |  |
| It is safer | 43 | 8.5 |
| It is less invasive | 19 | 3.7 |
| The surgeon recommended it | 30 | 5.9 |
| Faster recovery | 22 | 4.3 |
| Better outcomes | 26 | 5.1 |
| Not applicable | 387 | 76 |
| **Would you recommend robotic-assisted surgery to someone who needs surgery in the future?** |  |  |
| Yes | 126 | 27 |
| No | 53 | 11 |
| Not sure | 294 | 62 |
| **What improvements would you like to see in robotic-assisted surgery?** |  |  |
| Lower costs to make it accessible to more patients | 221 | 44 |
| Increased training for surgeons on robotic techniques | 251 | 49 |
| Development of new technologies to enhance precision | 238 | 47 |
| Improved safety measures to avoid errors | 256 | 50 |
| I don’t know | 129 | 25 |
| Others | 10 | 2 |

**Table S3: Sensitivity analysis using Firth penalized logistic regression with preference for a robot-assisted surgeon versus all other responses as the outcome**

| Factor | **aOR** | **95% CI** | **p-value** |
| --- | --- | --- | --- |
| Gender |  |  |  |
| Female | — | — |  |
| Male | 0.80 | 0.44, 1.43 | 0.500 |
| Age category |  |  |  |
| >=45 | — | — |  |
| 18-44 | 0.47 | 0.25, 0.87 | 0.016 |
| Nationality |  |  |  |
| Emirati | — | — |  |
| Non-Emirati | 0.65 | 0.36, 1.16 | 0.140 |
| Monthly income |  |  |  |
| <20,000 AED | — | — |  |
| >=20,000 AED | 1.17 | 0.66, 2.09 | 0.600 |
| Know someone who had RAS |  |  |  |
| No | — | — |  |
| Yes | 1.89 | 0.90, 4.02 | 0.091 |
| Understanding of RAS |  |  |  |
| Good | — | — |  |
| Limited | 1.10 | 0.63, 1.95 | 0.700 |
| Believes RAS is safe |  |  |  |
| Not sure | — | — |  |
| Yes | 2.48 | 1.39, 4.48 | 0.002 |
| No | 0.11 | 0.02, 0.36 | <0.001 |

aOR: Adjusted Odds Ratio, CI: Confidence Interval
